# Condom-use Skills Checklist: A Proxy for Assessing Condom-use Knowledge and Skills When Direct Observation Is Not Possible

**DOI:** 10.3329/jhpn.v27i3.3383

**Published:** 2009-06

**Authors:** B. Stanton, L. Deveaux, S. Lunn, S. Yu, N. Brathwaite, X Li, L. Cottrell, C. Harris, R. Clemens, S. Marshall

**Affiliations:** ^1^ Carman and Ann Adams Department of Pediatrics, Wayne State University School of Medicine, Detroit, Michigan, USA; ^2^ Office of AIDS, Bahamas Ministry of Health, Nassau, The Bahamas; ^3^ Behavioral Center, West Virginia University, Morgantown, West Virginia, USA

**Keywords:** Condom-use, Interventions, Knowledge, Scale, Skills, Bahamas

## Abstract

Because of the continued importance of correct condom-use in controlling the HIV epidemic and the limited availability of tools for assessing correct condom-use, methods for assessing condom-application skills, especially when direct observation is not feasible, are needed. Accordingly, in the context of a high-risk population (The Bahamas) for HIV, a 17-item scale—the Condom-use Skills Checklist (CUSC)—was developed for use among young adolescents and adults. The rationale and approach to developing the scale and some measures of internal consistency, construct validity, and criterion-related validity have been described. It is concluded that the scale offers a reasonable alternative to direct observation among older subjects and that further development may make it more useful among pre-adolescents.

## INTRODUCTION

Other than abstinence, protected sex remains the most effective prevention against acquisition of HIV and other sexually transmitted diseases (STDs) (1,2). Since incorrect condom-use significantly reduces the effectiveness of condoms, protected sex includes not only the use of condoms but also their correct use (3). It is estimated that condom-breakage/leakage or unintentional removal during pene-trative sex occurs at least once in a lifetime among 1-33% of persons ever using a condom, with higher rates of failure among adolescents than among older users (4-8). Strict control over manufacturing eliminates most structural flaws (9), leaving incorrect use as the major contributor to condom failure. Identified mistakes include completely unrolling the condom before application and using an oil-based lubricant (10,11). Moreover, there is evidence that youths with no or less knowledge, including that of correct condom-use, are less likely to use condoms than youths who have accurate knowledge (12). Therefore, both to reduce failure in condom-use and to augment condom-use, knowledge of correct condom-use is an important component of protected sex.

Despite our recognition of the importance of correct condom-use in prevention of the transmission of HIV and other STDs, few studies have assessed accuracy of condom-use skills in this context of disease-transmission studies. In one recent review of 45 studies assessing the effectiveness of condom-use against transmission of gonorrhoea and/or *chlamydia* published during 1966-2004, only two studies actually assessed the correctness of use, and these assessments were not comprehensive (2). In the first study conducted in California among 122 male patients returning to an STD clinic for follow-up and who had had sexual intercourse since the last visit, 75% had not used a condom, 17% had used a condom at least once, and 8% had used a condom at each sexual exposure. Users and non-users did not differ in rates of STDs at the follow-up visit but some condom-users who presented with re-infection with gonorrhoea reported that the condom was not put on until the man had already been exposed to vaginal fluids, suggesting that improper condom-use may have played a role in the failure of the condom to prevent infection (13). The second study, conducted among 1,122 female patients seen at a clinic for STDs in Alabama, during 1992-1995, involved two sub-studies: a cohort analysis among 919 women and a case-crossover analysis among 183 women. In the cohort analysis which only assessed condom-use, no protective effect was found for condom-use. By contrast, the case-crossover analysis of assessed self-reported condom-use and condom-breakage and slippage among the sample revealed protective effects of correct condom-use. Visits at which they were infected with gonorrhoea and/or *chlamydia* (n=228 visits) compared to visits in which they were not infected (n=743 visits) revealed a highly-protective effect of consistent condom-use without breakage or slippage. Sub-analysis also revealed that rates of STDs were lower in the intervals in which they reported consistent condom-use without breakage or slippage compared to those intervals in which they reported consistent condom-use but with breakage and slippage (14).

Since the promotion of both use and correct use of condoms are typically core components of HIV/STD-prevention interventions (15-17), some researchers conducting evaluations of these interventions have also attempted to assess the correctness of use as an intervention outcome (16). Examination of this aspect of intervention effect has generally relied on two approaches: self-reports of behaviour and direct observation. In the former, subjects are asked to describe their experience with condom-use during a defined period of time and to describe practices which might contribute to ineffectiveness of condom-use (e.g. using sharp instruments to open condom packages) or practices implying incorrect use (e.g. condom burst, dislodged) (18,19). These studies are limited by the inherent biases of retrospective self-report measures (18,19). Many studies have used condom-use self-efficacy as a proxy for actual skills (16). However, several studies have confirmed a discrepancy between participants' perceptions of their abilities and actual skills (20,21).

Direct observation of condom-application on penile models has been assessed by several researchers, using observed criteria, such as whether or not the subject squeezes air from the tip, unrolls the condom to the base of the penis, and leaves a space at the tip of the condom (15,22-24). Such direct observations may offer a more realistic assessment of actual application skills than do retrospective recalls of direct and indirect evidence of correct use but are not always feasible.

Despite the possible desirability of direct observation, this method of assessing correct condom-use is frequently not possible. Direct observation may not be feasible in large interventions in which many subjects may be completing an assessment measure at the same time, e.g. a classroom, or in settings with limited resources, including a paucity of trained study personnel, such as in developing countries. Moreover, in some settings, concerns have been expressed about allowing children to directly handle condoms (25), thus precluding direct observation of condom-use skills.

An example of a setting meeting several of these criteria, is The Bahamas where there is a substantial need for sexual risk-reduction efforts targeting adolescents and young adults. The Bahamas has the second highest annual incidence of AIDS in the Caribbean. Heterosexual activity is the predominant mode of transmission. An estimated 3.3% of adults are infected. After several years of declining incidence of HIV and prevalence of HIV/AIDS, the past three years have witnessed increases in both of these rates. This increase has been especially prominent among young adults. HIV is the leading cause of death among those aged 15-44 years for both males (76 per 100,000) and females (53 per 100,000) and is the leading cause of death among males of all ages. Among the inhabited islands, 84% of HIV-infected persons reside in New Providence (with 69% of the total population). In addition to the high rates of HIV infection among Bahamian adolescents and young adults, high rates of teenage pregnancy also provide evidence that a high proportion of youths is engaged in unprotected sex (26). The most current national data describing risk behaviours associated with HIV/STDs among high school students, The Bahamas Youth Health Survey 1998 (27) was conducted among ninth and eleventh grade students in Bahamian schools; 41% were sexually experienced, including 32% of those aged 13-15 years and 57% (70% of males and 41% of females) of those aged 16 years and older. The Bahamian Ministries of Education and Health have been committed to combating this national problem and have determined that the curriculum needs to be introduced in sixth grade before the rapid rise of sexual initiation begins. Moreover, they are committed to evaluating these intervention efforts to make certain that these are having an impact. However, because of concerns about allowing these young children to apply condoms to penile models in the school-setting and because of the resources that would be necessitated for an evaluation that includes over 1,000 youths and their parents (vide infra), they wanted an assessment measure that could be done by paper and pencil.

Accordingly, in the present paper, we describe an assessment tool designed as a proxy for observed condom-use skills. We present some psychometric properties of this instrument among pre-adolescents and adults in The Bahamas.

## MATERIALS AND METHODS

### General

The island of New Providence (the location of Nassau, the capital) was the site of the current research. The Bahamas has been an independent nation since 1973. Approximately 85% of all Bahamians and 91% of children attending public elementary schools are of African descent. School is compulsory through 16 years of age. Illiteracy rates are low (estimated at 2%), and English is widely spoken.

All the 26 elementary schools on the island of New Providence were invited in the summer of 2004 to participate in a randomized controlled longitudinal trial of a safe sex intervention being evaluated as part of the curriculum of the Bahamian Ministry of Education. Data for the present study were obtained from students of nine schools who first indicated willingness to participate in the study. There are two sub-studies described in this paper: the first is a paper-and-pencil questionnaire which was conducted among approximately 1,200 students and their parents, and the second was an small observational study conducted among a convenience sample of the adult subjects.

### Subjects

Seven hundred eighty-five youths (approximately two-thirds of the sixth grade students attending the nine schools) provided their assent and parental consent to participate and completed the measures described below at baseline. In addition, because of the strong evidence for the protective influence of parental communication and supervision on adolescents' risk and protective behaviour (28), their parents were also enrolled in one of the two complementary parent programmes—one emphasizing communication about prevention of HIV, including condom-use, and the other more general communication about goal-setting. Accordingly, 678 parents completed the measures described below at baseline. Subjects were asked to complete by pencil and paper a battery of measures, including the three measures, described below at baseline and then at follow-up to assess the intervention effect. Data used in this study were obtained from the baseline prior to administration of the intervention to youths and parents. The Human Investigation Committees of the Wayne State University and the Princess Margaret Hospital (The Bahamas) approved the study.

### Measures

The youths completed several measures, including the Bahamian Youth Health Risk Behavioural Inventory (BYHRBI)—a cultural adaptation of the Youth Health Risk Behavioural Inventory (25). The first section of the BYHRBI assesses demographic characteristics, and the second section assesses involvement in risk-behaviours, including sexual, drug-related and truant behaviours. The next section assesses perceptions of risk and protective behaviours, including perceived self-efficacy with regard to condom-use and intention to use a condom if they were to have sex. The final section assesses knowledge through a 20-item HIV/AIDS knowledge true-false questionnaire (including a 7-item transmission scale) which has been used in multiple settings across the globe. Psychometric properties of the scale have been described elsewhere and are adequate (25,29,30). For the present study, only the demographic and risk-behaviour subscale, condom-use self-efficacy and intention subscale, and the knowledge assessment portions of the BYHRBI were used.

The parents completed only the knowledge assessment of the BYHRBI.

The investigators developed a measure of condom-use skills—the Condom-use Skills Checklist (CUSC) consisting of 17 items as depicted in the figure. Items for inclusion in the scale were adapted from the instructions in the Focus on Youth with ImPACT: Adolescent HIV Prevention Program for African-American Youth with a Complementary Program for Parents curriculum (31), as well as the earlier edition of this publication (32). Criteria for modification of items included suggestions described by Lindemann and Brigham (16), e.g. each item must consist of a single step and be important in the prevention of STDs/HIV. We elected to include both correct and incorrect items. Of the 17 items listed, eight were correct steps, and nine were incorrect. Eight of the nine incorrect statements reflected an incorrect modification of one of the eight correct statements (such as “Wrap the used condom back in the foil to save for next time” is an incorrect version of the correct statement “Dispose of the used condoms” (Table 1). Accordingly, the subjects were told in the instructions that eight of the items were steps involved in correct condom-use while nine would not result in safe condom-use. The subjects were asked to circle the correct items. They received a score for accurate assignment of both correct and incorrect items, resulting in a total possible score ranging from 0 to 17. Both parents and youths completed this measure.

**Fig.**
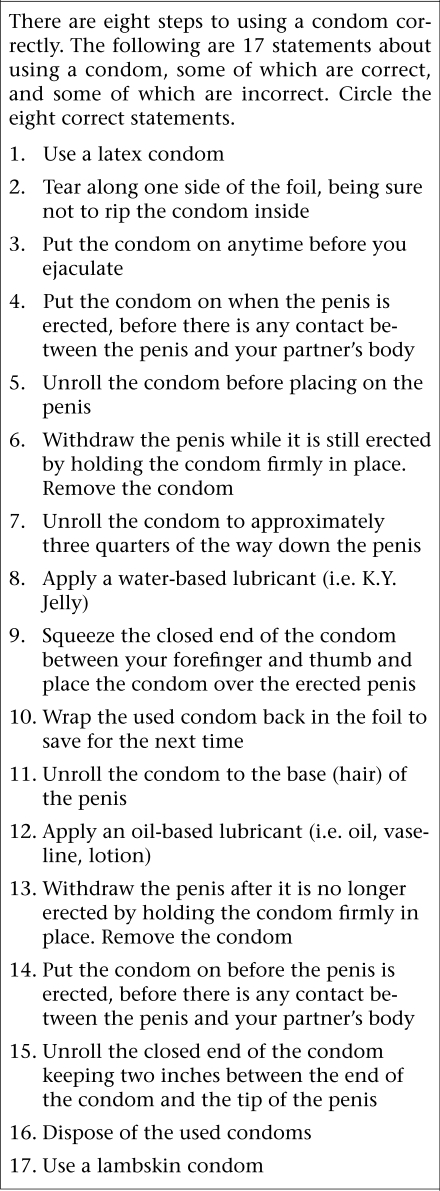
Condom-use Skills Checklist

**Table 1.** Sexual practices, intentions, and self-efficacy in condom-use among Bahamian youths

Description of youth	Overall	Male	Female
No. (%)	502 (100)	224 (44.6)	278 (55.4)
Age (years)	10.43 (0.73)	10.56 (0.76)	10.32 (0.69)
Ever had sex, no. (%)	15 (3.1)	12 (5.5)	3 (1.1)
Intention to have sex during the next six months∗	1.92 (1.16)	2.15 (1.25)	1.74 (1.06)
Condom-use intention during the next six months	3.16 (1.56)	3.30 (1.57)	3.05 (1.54)
Condom-use self-efficacy (I could put on a condom correctly)†	2.23 (1.34)	2.37 (1.45)	2.12 (1.23)

∗Response ranged from 1=Very unlikely to 5=Very likely

†Response ranged from 1=Strongly disagree to 5=Strongly agree

### Data collection

Data were obtained from youths and their parents at separate settings and times. The measures were administered to the youths at school during classtime by trained researchers who read out the questions loudly. As the administration of the CUSC followed the administration of the BHYRBI, several classrooms did not have a sufficient time to complete the measure, and therefore, the total number of students included in the present study was 502. Parents completed the measures at community settings at locations and times (typically evenings) convenient for the parents. The youth and adult versions of the knowledge portion of the BYHRBI and the CUSC were the same, except that the adult's knowledge scale had one extra question (21 compared to 20 in the youth's knowledge scale). Adults were not administered the risk or perceptions portion of the BYHRBI, and therefore, their risk and protective behaviours and self-efficacy/intentions with regard to condom-use were not assessed. All adults had a sufficient time to complete the CUSC. In addition to completing these measures, prior to completing the CUSC, a convenience sample of 24 adults was asked by one of the two study observers (either LD or SL) if they would first demonstrate the correct application of a condom on a plastic penile model. All but one of the adults (n=23), randomly approached, agreed to participate. (We did not conduct this exercise among youths as we were unable to obtain permission from the school system to do so.) The two study observers simultaneously observed the application of the condom, scoring for six tasks corresponding to six of the eight correct responses on the CUSC which could be verified by direct observation. There was 100% agreement between the scoring of the two study observers.

### Statistical analysis

We undertook several analyses to begin to establish the psychometric properties of the scale. We examined the internal consistency of the CUSC scale. We examined the correspondence of the CUSC with HIV knowledge (including transmission-knowledge and prevention-knowledge) to explore construct validity among both youths and adults. We examined the correlation between observed condom-use skills of the convenience sample of adults and the CUSC to explore criterion-related validity. (As noted above, we were not able to observe the youths placing condoms on a model; so, this analysis was restricted to the adults.)

To explore the internal consistency of the CUSC, we determined the Cronbach alpha of the scale overall and for the eight correct and nine incorrect items for both youths and adults. For assessing the correlation between the CUSC and the knowledge scale, we determined Pearson's moment correlation coefficients. We also explored the correlation with self-efficacy because, as noted in the introduction, these correlations have been examined by other researchers in the past (16,20,21) and with intention to use a condom and past sexual experience.

Agreement between the CUSC and the observed skills was calculated to compare the concordance between the CUSC and the actual condom-use skills (e.g. criterion-related validity of the CUSC as a proxy for observed skills). The numbers of participants who responded correctly on the CUSC and demonstrated correctly the same condom-use task plus those who responded incorrectly on the CUSC and demonstrated incorrectly the same condom-use task (numerator) were divided by the total number of participants (denominator).

## RESULTS

### General description of study populations

Of the 502 youths participating in the study, 224 (45%) were male. Table 1 depicts the baseline risk profile of the youths and their perceptions of condom-use self-efficacy and intentions. Sexual risk involvement was low among males and females. There was no gender difference in terms of sex intention, condom-use intention, and condom-use self-efficacy (Table 1). As shown in Table 2, adults had greater condom-use skills than did youths overall (p<0.001) and for all but two of the individual items. Male and female youths did not differ significantly in overall condom-use skills. Likewise, adults were significantly more knowledgeable about HIV than were youths (16.93 vs 11.97, p<0.001).

**Table 2.** Descriptive statistics (correct responses) for the Condom-use Skills Checklist

Condom-use skills	Overall correct responses		Youth correct responses	
	Adult (n=678)	Youth (n=502)	Male (n=224)	Female (n=278)
Use a latex condom	468 (69.3)	180 (52.0)∗∗∗	84 (54.9)	96 (49.7)
Tear one side, not to rip inside	489 (72.6)	210 (60.7)∗∗∗	100 (65.4)	110 (57.0)
Put the condom on when the penis is erected, before contact	534 (79.2)	224 (64.7)∗∗∗	101 (66.0)	123 (63.7)
Squeeze the closed end and place the condom over the erected penis	375 (55.6)	197 (56.9)	92 (60.1)	105 (54.4)
Unroll the condom to the base of the penis	482 (71.4)	211 (61.0)∗∗∗	108 (70.6)	103 (53.4)∗∗∗
Apply a water-based lubricant	97 (14.4)	113 (32.7)∗∗∗	48 (31.4)	65 (33.7)
Withdraw the penis while it is still erected by holding the condom firmly in place Remove it	312 (46.2)	186 (53.8)∗	92 (60.1)	94 (48.7)∗
Dispose of used condoms	519 (76.9)	207 (59.8)∗∗∗	96 (62.7)	111 (57.5)
Unroll the condom before placing on the penis	557 (82.5)	149 (43.1)∗∗∗	69 (45.1)	80 (41.5)
Apply an oil-based lubricant	612 (90.7)	225 (65.0)∗∗∗	101 (66.0)	124 (64.2)
Wrap used condom back in the foil to save for next time	669 (99.1)	241 (69.7)∗∗∗	97 (63.4)	144 (74.6)∗
Put the condom on anytime before you ejaculate	581 (86.1)	175 (50.6)∗∗∗	81 (52.9)	94 (48.7)
Put the condom on before the penis is erected, before contact	450 (66.7)	145 (41.9)∗∗∗	61 (39.9)	84 (43.5)
Unroll the closed end of the condom keeping two inches between the end of the condom and the tip of the penis	573 (84.9)	217 (62.5)∗∗∗	91 (59.5)	126 (64.9)
Unroll the condom to approximately three quarters of way down the penis	586 (86.8)	221 (63.7)∗∗∗	98 (64.1)	123 (63.4)
Withdraw the penis after it is no longer erected by holding the condom fully in place Remove condom	371 (55.0)	206 (59.5)	82 (53.6)	124 (64.2)∗
Use a lambskin condom	636 (94.2)	218 (62.8)∗∗∗	92 (60.1)	126 (64.9)
CUSC score (range 0-17), mean (SD)	12.29 (2.62)	9.58 (2.14)∗∗∗	9.76 (2.07)	9.44 (2.19)

Figures in parentheses indicate percentages

∗p<0.05

∗∗∗p<0.001; CUSC=Condom-use Skills Checklist; SD=Standard deviation

### Psychometric properties of CUSC

The Cronbach alpha for the CUSC overall among the 678 adults was adequate at 0.63 (0.80 for the correct answers and 0.47 for the incorrect answers). Among the youths, the alpha value was very low (0.08 overall, 0.07 for the correct answers, and 0.4 for the incorrect answers).

Table 2 shows that the overall knowledge of adults about HIV/AIDS correlated positively with the CUSC score (r=0.224, p<0.001) as did both subsets of transmission-knowledge (r=0.223, p<0.001) and general knowledge (r=0.169, p<0.001). Among youths, HIV transmission-knowledge correlated with the CUSC score (r=0.109, p<0.05); however, the overall HIV/AIDS-knowledge and general knowledge did not correlate with the CUSC score (r=0.086 and 0.043 respectively). Perceived self-efficacy regarding condom-use skills did not correlate with the CUSC score (r=0.050, p=NS). Neither intention to engage in sex nor condom-use intention correlated with the CUSC score. Likewise, prior sexual experience did not correlate with the CUSC score or HIV/AIDS-knowledge.

Comparison among 23 adults of the observed condom-use skills with the appropriate item on the CUSC revealed that agreement of responses (whether correct or incorrect) between the CUSC and the observed skills was generally high (four of the six exceeding 70% agreement).

## DISCUSSION

The CUSC is a scale assessing condom-use skills and knowledge that can be used in situations where direct observation is not feasible. In this setting in The Bahamas, using a cohort of pre-adolescents and a cohort of their parents, scores on the CUSC were significantly higher among adults than among youths. Likewise, the psychometric properties assessed among adults were reasonable, including the alpha value of the scale, correlation with knowledge, and agreement with observed condom-use skills. Therefore, the scale seems to be a reasonable choice for use among adults when direct observation of condom-use is not possible. Among youths, internal consistency of the scale was weak, and the correlation with knowledge was less strong, although there was a significant correlation with transmission-knowledge. Consistent with the literature regarding observed condom-use skills (20,21), the scale scores in the condom-use skill among the youths did not significantly correlate with the self-efficacy scores. Of some concern, these scores were also not higher among sexually-experienced youths nor among youths intending to have sex.

### Potential limitations

There are several potential limitations to this study. First, assessment of the CUSC against observed condom-application (gold standard) was not possible for the youths as noted under ‘Materials and Methods' section. While we were able to compare the measure with observations among a subset of adults, the number was small, and application of a condom on a penile model is itself a proxy for real condom-use. The assessment of condom-use skills through direct observations remains under development (16). The current scale does also not allow for any ‘weighting' of steps, i.e. currently, we are assuming that since each step is important, they are all valued equivalently while, in reality, some steps may be more important for effective condom-use than others. Second, it may be argued that the CUSC is still more a test of knowledge of skills than an assessment of actual skills. However, the focus of the CUSC on specific condom-use skills being demonstrated and practised in safe-sex prevention programmes should ensure that this assessment is more proximate to actual skills than the knowledge and self-efficacy assessments generally being used. Third, the psychometric properties that were assessed among the youths indicate that changes in the scale are needed among these young pre-adolescents.

### Implications of the study

There are several implications of this study. Further research is needed to address the limitations noted above, particularly for use among pre-adolescents. Importantly, this tool does offer an alternative even now, especially among older individuals, to augment approaches, such as general HIV-knowledge or condom-use self-efficacy testing which represent the measures of correct condom-use generally employed (16,18). While actual observation of condom-use skills rather than the use of a proxy tool may be preferred, such an approach may not be feasible for three reasons. First, as was the case in the present study, often school systems will not permit youths to handle condoms (25). Second, in large studies, such as the present one, direct observation of each subject would require substantial time and research resources, including trained personnel, which may not be available to many communities, especially those in developing countries. Finally, the methodology for observation is also still in the process of being developed (16) as the ability to apply a condom to a penile model may not accurately reflect application of a condom in a real-life situation.

In conclusion, given the apparent importance of correct condom-use in the effectiveness of condoms in the prevention of STDs and, therefore, the emphasis on this skill in HIV/STD-prevention programmes, it is important to assess condom-use skills, including condom skill-knowledge. The CUSC offers a reasonable alternative in such cases for older subjects but its use among pre-adolescents requires further adaptation.

## ACKNOWLEDGEMENTS

The authors thank the Bahamian Ministries of Health and Education and the families who participated in this study. The National Institute of Mental Health provided funding (Grant No. R01 MH069229) for this study.
